# Natural variation in non-coding regions underlying phenotypic diversity in budding yeast

**DOI:** 10.1038/srep21849

**Published:** 2016-02-22

**Authors:** Francisco Salinas, Carl G. de Boer, Valentina Abarca, Verónica García, Mara Cuevas, Sebastian Araos, Luis F. Larrondo, Claudio Martínez, Francisco A. Cubillos

**Affiliations:** 1 Millennium Nucleus for Fungal Integrative and Synthetic Biology (MN-FISB), Departamento de Genética Molecular y Microbiología, Facultad de Ciencias Biológicas, Pontificia Universidad Católica de Chile, Casilla 114-D, Santiago, Chile; 2 Broad Institute of MIT and Harvard, Cambridge, MA, United States; 3 Centro de Estudios en Ciencia y Tecnología de Alimentos (CECTA), Universidad de Santiago de Chile (USACH), Santiago, Chile; 4 Departamento de Ciencia y Tecnología de los Alimentos, Universidad de Santiago de Chile (USACH), Santiago, Chile

## Abstract

Linkage mapping studies in model organisms have typically focused their efforts in polymorphisms within coding regions, ignoring those within regulatory regions that may contribute to gene expression variation. In this context, differences in transcript abundance are frequently proposed as a source of phenotypic diversity between individuals, however, until now, little molecular evidence has been provided. Here, we examined Allele Specific Expression (ASE) in six F1 hybrids from *Saccharomyces cerevisiae* derived from crosses between representative strains of the four main lineages described in yeast. ASE varied between crosses with levels ranging between 28% and 60%. Part of the variation in expression levels could be explained by differences in transcription factors binding to polymorphic cis-regulations and to differences in trans-activation depending on the allelic form of the TF. Analysis on highly expressed alleles on each background suggested *ASN1* as a candidate transcript underlying nitrogen consumption differences between two strains. Further promoter allele swap analysis under fermentation conditions confirmed that coding and non-coding regions explained aspartic and glutamic acid consumption differences, likely due to a polymorphism affecting Uga3 binding. Together, we provide a new catalogue of variants to bridge the gap between genotype and phenotype.

Phenotypic variation between different individuals or populations is fundamentally polygenic, and depends on the interactions between genetic factors and the environment^1^. During the last decades, quantitative trait locus (QTL) mapping has been the most fruitful approach in the study of complex traits^2^. In this context, the majority of variants known to underlie polygenic traits alter protein structure and therefore protein-coding variants represent the primary targets in the search of causal polymorphisms^3^,^4^,^5^. A standard strategy for selecting candidates within a QTL is to predict the effects of SNPs on protein function and/or structure based on how conserved each residue is across a large number of species (Sorting Intolerant From Tolerant (SIFT) algorithm)^5^. However, in some cases non-synonymous SNPs do not account for the overall phenotypic variation. Alternative association signals, such as those implicated in variation in gene expression, have been shown to represent an important mechanism underlying natural phenotypic variation between individuals^6^,^7^,^8^,^9^. Indeed, nowadays studies in human cohorts focus their efforts on variants located within non-coding regions, likely affecting regulatory elements^10^,^11^.

A vast number of pioneer studies in structured populations in yeast have proven through the mapping of expression QTLs (eQTLs) that transcript abundance is subject to a defined genetic control^12^. Interestingly, these and other studies in model and non-model organisms have reported that a large proportion of the expression variance can be explained by polymorphisms near the encoded transcript, which presumably act in *cis* (*cis*-eQTLs). In contrast, genetically distant or unlinked eQTLs, presumably working in *trans* (*trans*-eQTLs), tend to explain less expression variance^12^,^13^. The thorough study of *cis*-eQTLs has been achieved utilising F1 hybrids and sequencing based methods (RNA-seq) for estimating allele-specific expression (ASE), where the *trans* effects are controlled for (the *trans* background is the same for the two alleles), allowing a direct estimation of the *cis*-effects on gene expression^14^,^15^,^16^,^17^,^18^. Nevertheless, currently there are a limited number of studies in yeast and other model organisms depicting the molecular mechanisms underlying natural expression differences between alleles^19^. For example, polymorphisms may affect expression through altered transcription factor (TF) binding^20^,^21^.

Even though ASE experiments have vastly expanded the number of alleles known to be differentially expressed between individuals in several organisms, such as humans^18^, Arabidopsis^14^, mouse^15^ and Drosophila^16^, the mechanisms by which expression differences between alleles impact phenotypic diversity is still unclear. Only recently, several studies have found examples where allelic expression variation has a substantial impact on natural phenotypic variation^22^,^23^. In an earlier study in yeast, it was shown that the differential expression of an aquaporin gene, *AQY2*, explained freeze tolerance differences across yeast isolates, where greater expression levels in the oak strain enhanced low temperature tolerance^24^. Such cases can provide important insights into the genetic bases of natural phenotypic diversity in the wild, for which, so far, little evidence exists.

Here, in order to test that allele-specific expression differences between isolates represents a tool to decipher the genetics underlying phenotypic diversity in natural populations, we evaluated ASE levels in a grid of six F1 crosses utilising an extensively studied set of natural isolates of *Saccharomyces cerevisiae*. We generated RNA-seq data in F1 hybrids and estimated genome-wide levels of ASE to assess the relevance of polymorphisms within non-coding regions upon gene expression variation and ultimately, phenotypic diversity. We found evidence for abundant genome-wide expression differences between alleles and we show that ASE can be explained by allele-specific differences in TF binding to *cis*-regulatory regions, providing direct evidence of how ASE can help understanding natural trait diversity.

## Results

### ASE differences across six F1 hybrids

**Figure 1:**
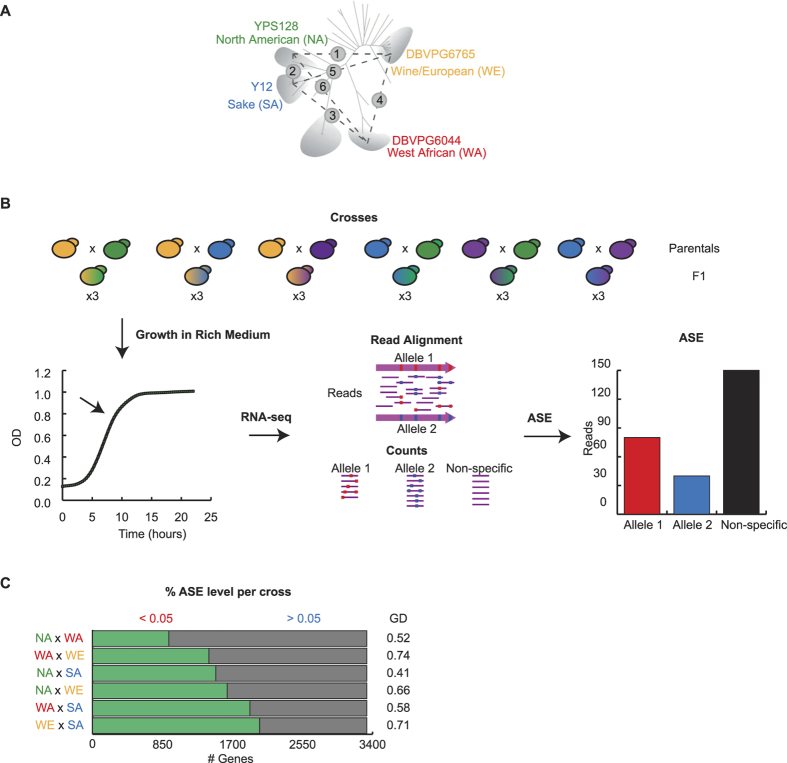
Allele-Specific Expression (ASE) between natural isolates. (**A**) The schematic tree highlights the four major geographic clusters previously described^25^. The four isolates (NA, YPS128; SA, Y12; WA, DBVPG6044 and WE, DBVPG6765) used to generate the F1 hybrids utilised in this study comprise a large fraction of the genetic variation in the species. (**B**) Schematic diagram representing the strategy followed to estimate ASE in budding yeast. Six F1 crosses were grown in rich media (YPD) in triplicates for RNA-sequencing in an Illumina HiSeq2500 platform. Reads were aligned against both genomes and reads specifically aligning to either parental background were considered for ASE estimation. ASE was estimated as the log_2_ ratio between the number of reads per gene for each parental background (red and blue). Replicates were treated independently throughout the process and ASE was estimated using edgeR (**C**) ASE levels per cross are depicted based on significance levels. Significant (green, FDR* *< 5%) and non-significant genes (grey, FDR > 5%) are shown together with the genetic distance (GD as %) between the strains based on Liti *et al.*, 2009.

In order to study the impact of individual alleles upon gene expression, we used a grid of six F1 hybrids derived from the cross of four representative founders of distinct geographic and ecological origins containing 64% of the *S. cerevisiae* SNPs so far described^25^,^26^ (Fig. 1a). We chose the YPS128 strain as representative of North America (NA), DBVPG6765 of the Wine/European (WE), DBVPG6044 of the West African (WA) and Y12 for Sake (SA), which present an average of one SNP every ~40 bp, with a pairwise density of one variant every ~200 bp^25^,^26^,^27^. To estimate ASE, F1 Hybrids were grown in triplicates in YPD (Fig. 1b) and we produced an average of ten million RNA-seq reads per sample (10.2 ± 2.3 million reads). In order to uniquely identify the parental alleles represented in the sequencing data, we aligned the reads to the appropriate heterozygous genomes (comprised of both parent transcriptomes) and extracted reads mapping to polymorphic regions based on previous resequencing studies^26^. We then quantified ASE using edgeR, excluding from the analysis those genes that were incompletely mapped within one or both parental genomes (see methods). We further defined a stringent set of genes to compare ASE between the six hybrids by only including genes for which at least 10 reads were obtained for each allele within at least one replicate per cross, yielding a set of ASE values per hybrid, but each hybrid having its own distinct set (hybrid-specific), To produce a set of genes for which ASE could confidently be assessed for all hybrids, we further restricted this set to those genes that had at least 10 reads per allele in all replicates of all hybrids, resulting in a final set of 3,321 genes (here after referred to as universally detectable ASE (UDA) genes). This set represents 50.2% of the annotated genes in the *S. cerevisiae* genome.

The number of genes showing significant ASE among UDA genes in F1 hybrids ranged from 923 (27.7%) to 2,024 (60.9%) genes (FDR 5%, corresponding to the NA × WA and the WE × SA crosses, respectively; Fig. 1c). Interestingly, across UDA genes we found that, on average, each gene showed significant allelic imbalance in three crosses (Fig. S1). The detailed results of mRNA abundance for each allele and cross are included in Table S1. In order to determine how widespread ASE is for each genetic background, we evaluated the number of genes exhibiting allelic imbalance per strain in at least a single cross. Overall, between 2,596 (78.2%) and 3,000 (90.3%) genes (in the NA and SA strains, respectively) showed ASE in at least one hybrid, demonstrating the ubiquity of ASE between natural isolates of *S. cerevisiae*. Altogether, these results suggest that ASE is commonly found in the budding yeast and set the ground to determine whether these expression differences could translate into phenotypic differences.

### Expression differences due to transcription factor binding site polymorphisms

**Figure 2:**
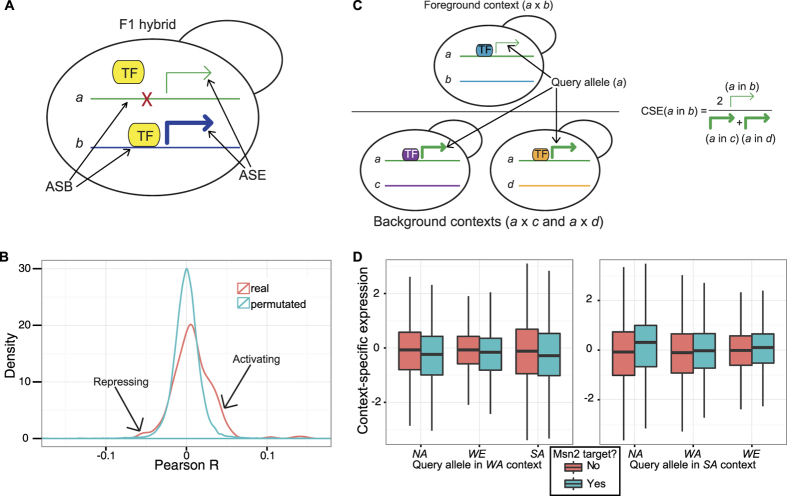
Motif analysis identifies mechanisms of ASE in both *cis* and *trans*. (**A**) Predicting ASE by ASB. Differences in binding sites can result in differences in TF binding and, consequently, differences in expression level. (**B**) Observed and expected (by gene-label permutation) Pearson R distributions for ASB and ASE. Shoulders to the right and left indicate activating and repressing TFs, respectively. (**C**) Context-specific expression (CSE) concept. When the green allele (strain ‘a’) shares a nucleus with the blue genome (strain ‘b’), its expression (green arrow) is in part governed by the blue TFs. CSE of the green allele in the blue context is equal to the ratio of the allele’s expression when paired with the blue TFs and that allele’s expression when paired with the purple (strain ‘c’) and yellow (strain ‘d’) TFs (as a background). (**D**) CSE distributions for predicted Msn2 targets and non-targets in the *trans*-context of WA and SA strains.

Because polymorphisms outside of the coding sequence are often assumed to be regulatory in nature, we next sought to explain ASE in terms of differences in allele-specific transcription factor binding (ASB; Fig. 2a). We first identified regions in each strain that are orthologous to the promoter regions of S288c, scanned these for all isolates using the expert-curated set of transcription factor (TF) motifs from the YeTFaSCo database^28^, calculated the probability of each promoter being bound by each TF in each strain, and calculated the differences in binding (ASB) between alleles (see **Methods** for details), yielding a number between −1 and 1 for each hybrid/TF/promoter combination representing the difference in TF - promoter binding between the two parental alleles. We observed that most of these values were close to 0 since the majority of TFs do not bind most of the promoters, and those that do are often conserved. Subsequently, we compared the predicted ASB to ASE in the six F1 hybrids, using the hybrid-specific ASE sets. Because a given set of polymorphisms will alter the predicted binding of multiple TFs, we cannot conclude causal links between changes in TF binding and changes in expression for specific genes. However, we can ask whether, across all genes, ASB correlates with ASE for a given TF, providing evidence that ASB caused ASE. We expect that an allele that is bound comparatively more by an activating TF should also tend to have a higher ASE. Conversely, an allele preferentially bound by a repressing TF should have a decreased ASE. Accordingly, the correlation between ASB and ASE is expected to be positive for activators and negative for repressors. Overall, we observed that ASE/ASB correlations differed significantly from what is expected by chance (K-S test of actual vs. gene-permuted Pearson Rs; *p-value *< 10^−8^), with many more apparent activating motifs 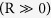 and only a very subtle enrichment for repressors (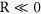), consistent with ASB of TFs having a predictable effect on ASE (Fig. 2b). We note that the correlation coefficients yielded in this analysis are expected to be quite small - if ASB and ASE had a perfect correlation (R = 1) then all ASE would be perfectly explained by the predicted ASB of a single TF. A more likely scenario is that, for a given TF, ASB is altered at only a few genes, explaining part of the ASE at only these genes - the vast majority of ASE (that at non-ASB genes) is attributable to other causes (e.g. other TF ASB, heritable chromatin state). Our results are consistent with the binding of many TFs being altered at many loci and affecting the expression of few genes each. Next, in order to find specific examples of TFs exhibiting a significant binding/expression association, we looked at the relationship between ASB and ASE for each motif individually (Table S2). We found that only a few TF motifs showed a significant correlation between ASB and global ASE after correcting for multiple hypothesis testing (Bonferroni), all of which represented motifs for zinc cluster TFs that have an activating effect (Spearman R = 0.05 − 0.08, *p*-value* *< 0.01). These included Sut1 and Ecm22, which are involved in regulating ergosterol uptake and synthesis, respectively^29^, Uga3, which activates GABA genes in response to gamma-aminobutyrate^30^, and the uncharacterized YLR278C. Altogether, this demonstrates that ASE can be explained on the basis of altered TF binding, but generally the binding of each TF is altered at only a few genes.

We next sought to take advantage of the shared *trans* environment in the F1 hybrids to identify potential differences in TF activity between strains. In general, if one strain has an allelic form of a regulatory protein (TF or otherwise) with an altered function, when this alternate form is present (e.g. in the hybrids containing this allele), the targets of this protein may be differentially regulated. Note, however, that if the regulatory pathway ends with transcriptional changes via a particular TF with a known motif, we may see coordinated changes in the expression of that TF’s targets when the alternate allelic form is present. In particular, if a TF suffers from a loss of function mutation in one background, we expect hybridisation with a functional TF background to (partially) rescue TF-target regulation of the null background alleles, while the TF’s targets in the functional TF background may decrease in expression due to haploinsufficiency. With this in mind, we sought to identify TFs that appear to be more or less active in one strain as compared to the other three by looking at how the expression of that TF’s targets change in the different hybrid contexts. Thus, we defined the context-specific expression (CSE) of an allele (*a*) in a hybrid context (*a* × *b*; foreground) as the allele’s expression level in the *trans*-environmental context of *b*, divided by the average expression level of that same allele in the other two F1 hybrid contexts (*a* × *c* and *a* × *d*, *c* and *d being* the background genetic contexts; Fig. 2c). A relatively high CSE of an *a* allele in the context of *b* indicates that the *trans*-factors of *b* are activating *a* more than the *trans*-factors of *c* and *d*, and a low CSE indicates the opposite. Thus, the CSE can be thought of as the contribution of the *trans*-environment on expression of an allele (see **Methods** for further explanation).

When comparing the CSE between target and non-target genes for each TF as defined by motif instances (see **Methods**), we found that several TFs had a significant association with CSE (FDR q ≤ 0.1; Table 1; all results in Table S3). For example, the targets of Sum1, a transcriptional repressor involved in repression of sporulation-specific genes during meiosis^31^, are upregulated in the WA context and downregulated in the NA context (*q *< 10^−6^ and *q* = 0.009, respectively), implying that Sum1 is less repressive in WA and more repressive in NA. We found that Ste12, a transcriptional activator involved in mating and pseudohyphal growth^32^,^33^, activates its targets more in WA and less in SA (q = 0.1 and q = 0.08, respectively), while Phd1, a transcriptional activator regulating pseudohyphal growth^34^, activates its targets less in WE (q = 0.07). Finally, Msn2/4 appear to activate their targets more in SA and less in WA (*q* = 0.0006 and *q* = *0.009,* respectively for the Msn2 motif; Fig. 2d). We note, however, that there are several related TFs that bind very similar motifs to Msn2 and Msn4 (e.g. Usv1 and Rgm1), and so we cannot be certain that Msn2/4 are the factors mediating the differences in *trans*-activation with this technique. Altogether, these results demonstrate that the nature of *trans*-acting factor variants can be inferred by differential regulation of their predicted targets, taking advantage of the shared *trans* environment in F1 hybrids.

### Identification of candidate genes underlying natural phenotypic variation for oenological traits

**Figure 3:**
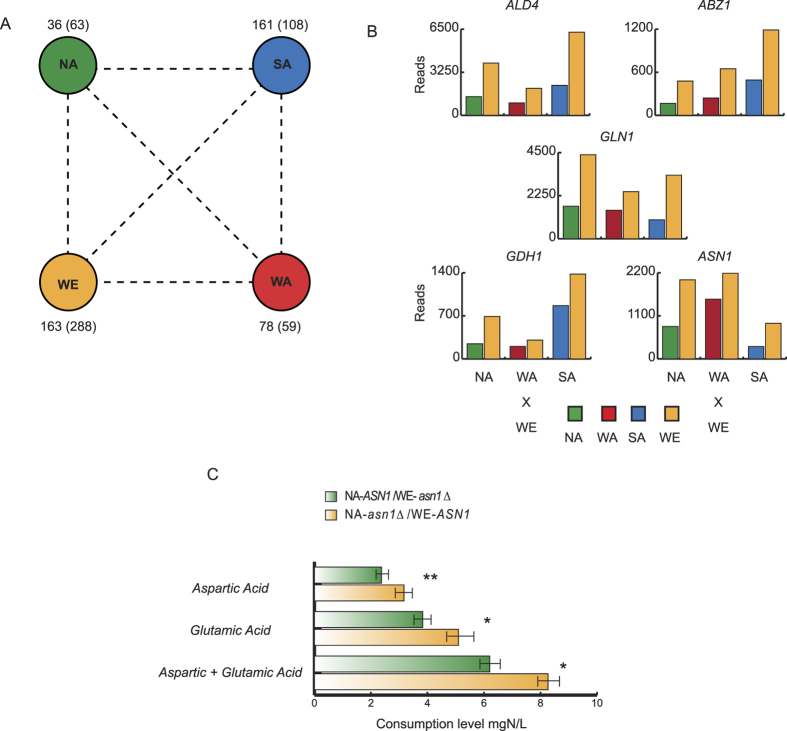
Directional Allelic Selection. (**A**) Diagram representing the number of alleles maximally expressed and (minimally expressed) on each genetic background based on ASE levels (FDR* *< 5%) on the three hybrids where the corresponding parental strain is involved. (**B**) ASE levels in the set of maximally expressed alleles in the WE background for the ‘carboxylic acid biosynthetic process’ GO term. The number of reads for each allele in the three crosses involving the WE strain is shown for *ALD4, ABZ1*, *GLN1*, *GDH1* and *ASN1*. (**C**) Amino Acid consumption levels (mgN/L) for aspartic acid, glutamic acid and the sum of both are depicted for NA-*ASN1*/WE- *asn1*Δ (green) and NA-*asn1*Δ/WE-*ASN1* (orange) reciprocal hemizygotes. Significant differences were found for aspartic acid (*p*-value* *< 0.004), glutamic acid (*p*-value* *< 0.04) and the sum of both (*p*-value* *< 0.02).

In order to find evidence of signatures of directional allelic selection in the different parental strains that could shed lights into natural phenotypic adaptation, we looked at alleles over (maximally)- or under(minimally)-expressed in one genetic background compared to any other. Genome-wide, we observed that the WE strain contained the greatest number of maximally/minimally-expressed alleles with 163 and 288 genes, respectively (Fig. 3a). Interestingly, the NA and WA strains, the two non-industrial isolates, showed the lowest number of maximally (36) and minimally expressed alleles (59), in agreement with the lowest genome-wide ASE levels found in these two genetic backgrounds (Fig. 1c).

In order to test for directional allelic selection, we searched for functional categories or pathways that are significantly enriched in the sets of maximally- and minimally-expressed genes in each strain, using the gene functional classification assigned by the Gene Ontology Consortium^35^ and the Kyoto Encyclopedia of Genes and Genomes (KEGG) database for pathways maps and molecular interactions^36^. We identified 17 significant categories at a 10% FDR for genes under ASE (Table S4), the majority of which represented categories enriched within minimally-expressed alleles. For example, we found a four-fold enrichment of the ‘Amino acid transport and metabolism’ term for alleles maximally-expressed in the SA background (FDR = 9.9%). Among genes maximally-expressed in the WE background, the ‘carboxylic acid biosynthetic process’ GO term was found (FDR = 8.5%; fold enrichment =2.3). Interestingly, most of these genes, such as *ABZ1*^37^, *ALD4*^38^, *GDH1*^39^, *GLN1*^39^ and *ASN1*^40^, have been previously reported to be involved in the fermentation process, in agreement with the origin of the strain over expressing these alleles (Fig. 3b). These results demonstrate the presence of a robust directional allelic selection in the different genetic backgrounds for genes involved in related functions or pathways.

Next, to demonstrate that the genes responsible for these functional enrichments underlie phenotypic differences between natural isolates, we performed a reciprocal hemizygosity assay on several candidate genes. In particular, although ASE was estimated in optimal growth conditions, we focused on oenological phenotypes, assuming that *cis*-effects on ASE are more likely to change in magnitude rather than in direction^14^. Thus, we looked for candidate genes within the set of maximally-expressed alleles in the WE background that could be involved in processes related with wine fermentation. We selected the candidate genes *GDH1*, a glutamate dehydrogenase and *ASN1* an asparagine synthetase that catalyses the synthesis of L-asparagine from L-aspartate, for the reciprocal hemizygosity assay. The selection of these genes was based on the fact that glutamine and aspartic acid are utilised as nitrogen sources during the fermentation process. For these two genes we found the largest expression differences between the WE and NA strains, and therefore the WE × NA hemizygotes were chosen for the phenotypic assay. Reciprocal hemizygotes were fermented at 25 °C during 21 days in MS300 (see methods) and fermentation kinetics (CO_2_ output) together with phenotypes of oenological interest were measured. Since *ASN1* is involved in asparagine synthesis from aspartate, we evaluated aspartic acid and glutamic acid assimilation profiles in the reciprocal hemizygotes. As hypothesised, differences were observed between allelic variants, where the WE-*ASN1*/NA-*asn1*Δ hemizygote showed higher consumption levels of both amino acids (*P *< 0.05, ANOVA), with respect to the WE-*asn1*Δ/NA-*ASN1* hemizygote (Fig. 3c, Table S5a). Moreover, this result is in agreement with greater expression levels of the WE allele. Analyses of *GDH1* nitrogen assimilation profiles in the WE × NA hemizygotes did not show evidence of differences in any of the sources evaluated (Table S5b), suggesting a more complex regulation pattern or mild phenotypic consequences in this case.

### Phenotypic differences between *ASN1* alleles are caused by polymorphisms within coding and non-coding regions

**Figure 4:**
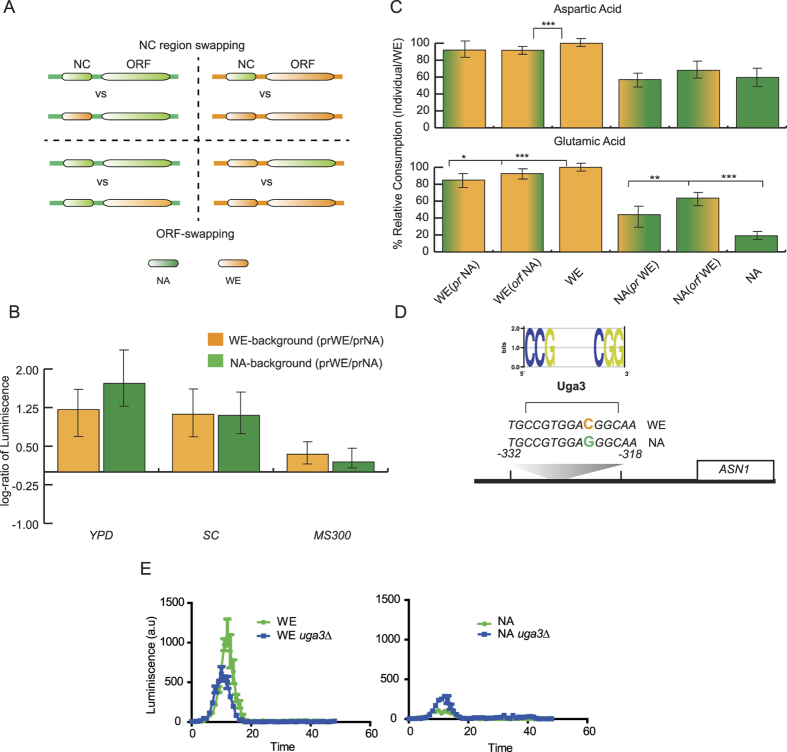
Promoter allele swaps for *ASN1*. (**A**) The swap strategy for promoters (top blue) and ORF regions (bottom red, either *ASN1* or Luciferase) are shown. Each promoter and ORF was swapped in the opposite genetic background, generating four different combinations. The strain background is shown as an orange (WE) or green (NA) rectangle. (**B**) Luciferase expression levels on the different genetic backgrounds were estimated as the log2 ratio of the strains carrying the *ASN1*^*WE*^ promoter (prWE) and *ASN1*^*NA*^ promoter (prNA) in YPD, Synthetic Complete media (SC) and fermentation must (SWM). **(C)** The relative consumption of aspartic acid and glutamic acid are plotted for each background (WE – green; NA – orange), normalised such that WE represents 100% consumption. Mutants containing swapped promoters (WE prNA and NA prWE) and ORF (WE orfNA and NA orfWE) are shown with hybrid colours. **(D)** The sequence divergence between WE and NA strains between −332 and −318 bp from the *ASN1* ORF together with the predicted ASB differences due to the C/G polymorphism are depicted. (**E**) Luciferase expression levels were estimated as luminiscence (a.u) levels in the WE (green) and NA (orange) backgrounds as well as the corresponding *uga3*Δ mutants grown in Synthetic Complete media (SC).

Initially, in order to validate *ASN1* allelic expression differences, we performed an allele swap strategy of the native promoters in the parental strains (700 bp upstream the ORF) and inserted immediately downstream a luciferase reporter replacing the original *ASN1* locus (Fig. 4a). The luciferase expression levels driven by each promoter on each genetic background were then measured by high-throughput phenotyping in YPD (ASE condition), synthetic complete media (SC) and synthetic wine must to differentiate the strength of the two promoters under several environments. In all three conditions, the *ASN1*^*WE*^ promoter exhibited greater expression levels, independently of the genetic background (either when placed in the original WE background or in the NA strain, Fig. 4b and Fig. S2), validating our original ASE results.

Subsequently, to determine whether the phenotypic differences between *ASN1* hemizygotes are due to polymorphisms within either coding or non-coding regions, we repeated the allele swap strategy of the promoters in the parental strains, but this time utilising the original *ASN1* ORF. Based on this approach, we reconstructed all the possible combinations in the parental backgrounds by swapping either the ORF or the regulatory region (Fig. 4a) and evaluated the nitrogen sources for which we previously found significant differences in *ASN1* (Fig. 3c). Initially, we examined the assimilation profiles of *ASN1* in parental strains and found that the NA strain had 41% and 81% lower assimilation levels of aspartic and glutamic acid, respectively (Fig. 4c). Subsequently, we evaluated the nitrogen assimilation profiles of the haploid mutants carrying the swapped alleles. When we incorporated the *ASN1*^*NA*^ non-coding region into the WE background, we found a significant difference for glutamic acid, with a 13% decrease in the consumption levels, in agreement with the tendencies observed in the parental strains (Fig. 4c). Similarly, swapping the *ASN1*^*WE*^ regulatory region into the NA background showed a significant 25% increase in the amount of glutamic acid consumed (*p*-value* *< 0.05, ANOVA), but no significant difference in the case of aspartate. The equivalent experiment swapping the coding regions showed a significant 8% decreased assimilation level for both amino acids in the WE background, while a 46% increase in glutamic acid was estimated when the WE ORF was incorporated into the NA background (Fig. 4c).

Finally, with the aim of determining which polymorphisms could be underlying the phenotypic differences observed, we compared sequence divergence between the WE and NA backgrounds in the regulatory region within the 700 bp immediately upstream of the ATG and found a total of five polymorphisms. Subsequently, we evaluated binding differences for each polymorphism in the non-coding part and found a C/G (WE/NA) SNP at position −323 located within a Uga3 binding site. The presence of this SNP would result in comparatively less TF binding in the NA strain (Fig. 4d, position weight matrix score decreased by 45% in the NA background). In order to determine the influence of Uga3 upon *ASN1* expression, we generated *uga3* deletions for those strains carrying the luciferase construct and estimated expression levels in YPD, SC and SWM. When we compared the WE background against the WE-*uga3*Δ knockout, we observed a significantly greater luciferase activity in the WE strain carrying an active version of *UGA3* when grown in YPD and SC media (*P *< 0.05, Mann-Whitney U test, Fig. 4e), and confirming the higher expression levels of *ASN1*^*WE*^ promoter compared to *ASN1*^*NA*^ promoter (Fig. 4e). The same pattern was not observed in the NA background, where a greater expression level was estimated in the knockout strain (Fig. 4e). Thus, our results suggest an active role of Uga3 in regulating *ASN1* expression in the WE background. Interestingly, Uga3 has been implicated in nitrogen catabolism and represents a potential candidate to underlie the phenotypic differences observed between allelic variants. On the other hand, all the polymorphisms identified in the coding portion of *ASN1* represent synonymous SNPs and therefore no obvious candidates arise from this region.

Overall, these results validate the role of *ASN1* upon the nitrogen consumption profile in these isolates and demonstrate that both, coding and non-coding regions support natural phenotypic variation between these two genetic backgrounds.

## Discussion

During the last decade, many studies have aimed to decipher the natural variants underlying complex traits. However, candidate QTLs have been usually biased towards SNPs that can directly affect protein function. While many of these studies were successful using this strategy and have finely tuned our understanding of the molecular bases underlying complex traits, such approach has neglected the contribution of non-coding regions. Thus far, much of our knowledge about polymorphisms within regulatory regions and their downstream effects on variation in gene expression derives from large-scale studies devoted to find expression QTLs and, lately, differences in allele-specific expression^14^,^15^,^17^. However, evidence on how causal non-coding variant affect natural gene expression variation and phenotypic diversity is still limited, probably because trait differences due to transcript abundance changes are difficult to demonstrate and validate. Recently, a few ambitious efforts have extended our understanding on the molecular mechanisms underlying transcriptional variation, providing rich evidence on how natural *cis*-variants influence downstream gene expression^9^,^20^,^22^.

Here, we initially sought to investigate the extent of ASE across four natural isolates of *S. cerevisiae*. While our experimental design extends previous reports on ASE in laboratory yeast strains^41^, we found that our analysis of gene expression between four wild strains reveals a substantial increase in the number of alleles differentially expressed (Table S1). Previous microarray analysis between lab strains have reported over 20% of the genes evaluated exhibiting differences in expression levels due to local (likely in *cis-*, but no necessarily) factors^42^. In our study, ASE levels range between 28% up to 61% depending on the cross (Fig. 1c), with almost 97% of the genes analysed showing ASE in at least one cross. Interestingly, ASE levels per cross do not correlate with the genetic distance between strains (Spearman test, R = 0.08, *p-*value = 0.87), in support of the idea that phenotype may be better predicted by ecological niche than by genetic relationship. The increased power to detect transcript abundance variation is not solely due to the greater resolution when using high read counts, but instead to the utilisation of genetically distant strains, a controlled environment and the easy manipulation of individual yeast samples. Furthermore, the wide range of genes exhibiting ASE could be explained by the extensive number of private polymorphisms between the strains, in contrast with other reports between closely related individuals^41^. Similar observations were reported in mice, where the utilisation of inbred individuals in a controlled environment led to high levels of transcript abundance variation^15^.

By predicting TF binding using motifs previously characterised in the laboratory strain S288c, we were able to further enlighten our understanding of the mechanisms behind ASE. We demonstrated at the genome-wide level that the differences in predicted TF binding due to *cis*-regulatory variation affect transcription in a predictable way. Interestingly, we found that most significant associations between ASE and ASB were positive, indicating that most TFs with significant associations function as activators. Recent work indicates that activators and repressors are comparable in number and have similar numbers of target genes^43^, but a tighter association between binding and transcriptional output among activators, or a greater context-dependence of repressor activity could explain this observation. However, we note that since our analysis was limited to growth in rich media, it is possible that a greater enrichment among repressors may be evident in less transcriptionally active conditions.

The shared *trans*-environment within F1 hybrids allowed us to shed some light on the *trans*-determinants of expression variation by identifying TFs whose targets are up- or down-regulated in a particular hybrid context. For instance, we observed that the positive regulators of invasive growth Ste12 and Phd1^44^ appear to be more active in WA and less active in WE, respectively, which could explain why WA exhibits the greatest amount of invasive growth of these four strains and why WE does not exhibit invasive growth^45^. In addition, many of the motifs predicted to have allele-specific activity correspond to the stress-response element bound by Msn2/4^28^. In particular, we found WA to have a deficient stress response, consistent with previous phenotypic characterization^27^, while SA had a more active stress response. We detected this in spite of our ASE data being generated in comparatively stress-free growth conditions and so the observed differences may be exaggerated under stress. It is important to note, however, that, our approach to identify *trans*-determinants of ASE cannot distinguish between differences in the regulating TF, one of its specific cofactors or an upstream regulator as all would yield changes in the expression of the TF’s targets. We further noticed that, although we found several promising examples of differential *trans*-regulation using CSE, we had a limited amount of data to use in our analyses: only four parental strains and three F1 hybrids containing the same queried strain, leaving us only a single strain to use as foreground and two for background. With more hybrids, our ability to dissect differential *trans*-regulation would be likely improved. Moreover by combining this approach with eQTL data, we may be better able to identify the exact nature of the differentially regulated pathways. For instance, *REX4*, which interacts genetically with *MSN2* and *MSN4*^46^, lies within a *trans*-eQTL found to regulate Msn2/4-dependent genes^42^.

Based on our ASE dataset, we found signatures of directional allelic selection for several gene ontology terms significantly enriched in the different parental backgrounds, where sets of genes with related function are either over or under-expressed (Table S4, Fig. 3). This strategy has previously proven fruitful in crosses between *Saccharomyces* species, revealing how *cis*-regulatory variation between species influences pathway divergence^47^,^48^. The WE background exhibits enrichment for several gene ontology terms among over-expressed genes, out of which the ‘carboxylic acid biosynthetic’ term contains several genes (*ABZ1*, *ALD4*, *GLN1*, *GDH1* and *ASN1*) previously associated to oenological phenotypes^37^, in agreement with the genetic and phenotypic clustering of this strain with others isolated from fermentation musts^25^,^27^. Thus, in order to demonstrate that expression differences between allelic variants could underlie natural phenotypic variation between strains, we generated reciprocal hemizygotes among backgrounds with the greatest absolute ASE log_2_ ratio in the candidate genes *GDH1* and *ASN1*. Phenotypic differences between hemizygotes were observed in *ASN1*, but not for *GDH1* (Table S5, Fig. 3). *ASN1* encodes for an asparagine synthetase and allelic variants show significant differences for nitrogen assimilation preferences, in agreement with greater expression levels of the *ASN1*^*WE*^ allele and suggesting a greater capacity to assimilate these two amino acids. Certainly, previous transcriptome studies have also reported variation in expression levels in *ASN1* under fermentative conditions, highlighting the importance of adequate nitrogen levels during the course of the fermentation process^40^.

Do coding or non-coding polymorphisms explain the phenotypic differences observed between allelic variants? To answer this question, we designed a promoter and ORF-swap strategy for *ASN1* where we exchange one or the other region with the alternative allelic variant in the parental backgrounds (Fig. 4a). After reconstructing the mosaic parental backgrounds carrying alternative versions of regulatory regions, the promoter allele swap strategy for *ASN1* in the NA background demonstrates that the non-coding region is partly responsible for the phenotypic variation between these two backgrounds, where the WE regulatory region increases the glutamic acid uptake in the NA background (Fig. 4c). Accordingly, the opposite effect is observed in the WE background, where the presence of a NA promoter decreases glutamic acid assimilation. A similar observation was also obtained in both backgrounds when swapping the ORF. Part of this phenotypic divergence could be likely explained by ASB of Uga3, which could preferentially bind the WE regulatory region (Fig. 4d,e). We further note that Uga3 was one of the few TFs to have a significant association between changes in its binding sites and changes in expression of the associated gene (Table S2), indicating that regulation by this TF may have rapidly diverged among these strains. These results demonstrate that the non-coding region would also be responsible for the quantified phenotypic differences between isolates.

In conclusion, our results demonstrate that non-coding genetic variation has widespread effects on gene expression levels, resulting in phenotypic variation with functional consequences. We identified thousands of genes under ASE and provided evidence for how altered TF binding and activity would impact expression levels in each genetic background to shape the phenotypic landscape. The ASE dataset here provided can be used as a tool to undercover many variants underlying other phenotypes of industrial or medical interest, assisting the identification of functional regulatory polymorphisms underlying complex traits. Finally, we demonstrated new paths to bridge the gap between genotype and phenotype diversity, by integrating allele-specific expression levels with natural trait variation.

## Methods

### Strains, culture conditions and RNA extraction

The F1 hybrids used in this study were previously generated from crosses between haploid strains from different geographic origins: YPS128 (North American, NA), DBVPG6044 (West African, WA), Y12 (Sake, SA) and DBVPG6765 (Wine ⁄ European, WE). These strains, together with the F1 hybrids, have been described in detail elsewhere^49^. Each F1 hybrid was grown in triplicate in 5 mL of rich yeast peptone dextrose (YPD) media at 28 °C up to mid-log phase (OD_600_,~0.8). Cultures were harvested by centrifugation and cells were treated with 2U of Zymolyase for 30 min at 37 °C. RNA was extracted utilising the E.Z.N.A. Total RNA Kit I (OMEGA) according to the supplier’s instructions. RNA samples were then treated with DNase I (Promega) to remove genomic DNA traces and total RNA was recovered using the GeneJET RNA Cleanup and Concentration Micro Kit (Thermo Scientific). RNA integrity was confirmed using a Fragment Analyzer.

### RNA sequencing and ASE data analysis

The RNA-seq libraries were constructed using the TruSeq RNA Sample Prep Kit v2 (Illumina). Briefly, mRNA from 1*u*g of total RNA was enriched by mRNA purification magnetic beads. Enriched mRNA was eluted and fragmented at 94 °C for 5 min. The double stranded cDNA was acquired by RT-PCR using above fragmented mRNA, followed by end repair, single A base adding and Adapter index ligation. The ligation product was amplified by PCR. The size of the end product was around 260 bp. The sequencing was conducted on HiSeq 2500 (Illumina).

In order to map RNA-seq reads to the hybrid transcriptomes, we created six separate reference transcriptomes, each containing the union of the parental transcriptomes (hybrid transcriptome). ORF locations were identified in each strain by mapping the ORF annotations from S288c to each parental strain using UCSC’s liftOver tool. The custom chain files required were created by mapping homologous sequences between each strain’s genome^26^ and the S288c genome (R64, sacCer3), using Blat^50^. This yielded ORF sequences for each strain, for most ORFs – incompletely mapped or fragmented ORFs were excluded from subsequent analysis (326–509 per parental strain). Reference transcriptomes were created for each hybrid by combining the ORF sequences from each parental allele – UTR sequences were excluded because their lengths were less likely to be conserved. RSEM^51^ was used to align reads to the appropriate hybrid ORFeome using Bowtie^52^, with reads subsequently filtered for those that prefer one parental allele over the other (i.e. the read contains one or more bases that differentiate the alleles). Overall, nearly 15% of the reads corresponded to allele-specific reads. These reads were then summed for each allele and used in subsequent analyses. In some cases, ORFs were found to contain Ns (i.e. bases that are undefined in the genome reference), or the ORF was missing from one or both parental reference genomes, and/or there were fewer than 10 reads for all replicates of one allele and these ORFs were excluded from all subsequent analyses for the affected hybrids to avoid mapping biases. Overall, for the ASE analysis, we excluded 2,259 genes due to the absence of polymorphisms, missing ORFs or undefined bases in the genome.

ASE for each gene was calculated by comparing the allele-specific counts between parental alleles for each replicate of each hybrid. Since within most hybrids the number of genome-wide reads differed between both parental alleles, we TMM-normalized the expression data prior to further manipulation. We next used edgeR to calculate ASE log-ratios and p-values, comparing the three replicates of each allele. Practically speaking, the two alleles for each gene were compared as if they were the same gene in two different conditions, each having three replicates. We found this approach to work well in our context because the two parental haplotypes are fully known and so we did not need to concern ourselves with incomplete linkage or multiple possible polymorphisms. Further, our approach has the advantage that even large polymorphisms that may not align to the reference (S288c) genome will still be detected (by mapping correctly to that polymorphism in the parental strain’s allele). This provided us with a set of ASE values per hybrid, where the genes for which we had ASE values varied based on what genes were present and abundant in that particular hybrid. To yield a set of genes that could be compared across hybrids (the UDA set), we further eliminated genes containing fewer than 10 reads per allele across replicates in at least one cross were discarded from the analysis (in total, 1,027 genes). *P*-values were corrected for multiple hypothesis testing using the q-value package^53^. The q-values utilised represent the minimum FDR at which each individual ASE event is significant.

### Scanning orthologous promoter regions for transcription factor binding sites

Promoter regions were taken as the region from −250 to −50, relative to the transcription start site in S288c, and orthologous promoter regions were mapped from S288c, as described above for ORFs. Where promoter sequences were incompletely mapped or contained Ns, the promoters were excluded from subsequent TF-binding analyses. TF binding probability was calculated using the GOMER model^54^, with allele-specific binding as the difference between the probability of binding for one parental allele and the other. Particularly, for *ASN1* we analysed the regulatory region taken from −700 to the ATG.

### Calculating context-specific expression (CSE)

Here, we will refer to the parental strain whose TF activities are being tested as the “foreground strain”, the other parental strain in the F1 hybrid as the “query strain”, and the two other F1 hybrids with the query strain as a parent as the “background strains”. Thus, in the query x foreground hybrid strain, the query’s genes are being expressed in the context of the foreground’s *trans*-acting factors, as well as its own (but these are also present in the two other background strains). We calculated a context-specific expression (CSE) for each query gene in each F1 hybrid context by (first, TMM normalizing the expression data, and then) calculating the expression ratio between expression in the foreground context and background contexts. For example, the CSE of the query *WE* allele in the *SA* foreground context (F1 *SA* × *WE*) is taken as CSE(*WE*,*SA*) = (2 × Expr(*WE*,*SA*))/(Expr(*WE*,*WA*) + Expr(*WE*,*NA*)), where Expr(*WE*,*SA*) is the expression of the *WE* allele in the *WE* × *SA* hybrid. This way, if the gene is activated more in the foreground context than in the background contexts, a positive CSE results and we can infer that there may be some *trans*-acting factor activating the gene in the foreground that is not present in the background strains.

To compare CSE between TF targets and non-targets, we defined a TF’s targets as genes with at least half the maximum observed motif score, unless these numbered fewer than 50 or more than 10% of the total potential targets, in which cases the top 50 or the top 10% of genes were taken as targets, respectively. To test if targets and non-targets have significantly different CSEs, we applied a Student’s T-test and ensured that, for a given context, the same relationship between TF targets and CSE held for all three possible query parents. We then used a Benjamini-Hochberg FDR (alpha = 0.1) correction to define significant hits.

### Directional Allelic Selection

To assess directional allelic selection on each genetic background, we used the set of alleles maximally- or minimally-expressed in all three hybrids involving a common parental strain (or “query strain”). Later, these eight sets of genes (two for each strain, one containing over-expressed alleles, while the other containing under-expressed alleles) was used in the DAVID Bioinformatics Resource^55^ to test for a significant enrichment in gene ontology (GO) terms and/or pathways in the Kyoto Encyclopedia of Genes and Genomes (KEEG). We selected categories with a significant over-representation of ASE events utilizing a FDR* *< 10%.

### Generation of reciprocal hemizygotes, promoter swapping and phenotyping

Reciprocal hemizygotes for each candidate gene were generated as previously described^3^,^56^,^57^. Briefly, the gene *URA3* previously deleted in the parental strains^58^ was used as a selectable marker. Haploid versions of the parental strains containing opposite Hygromycin B and Nourseothricin cassette resistances were used to delete each target gene and construct all possible combinations of single deletions. Next, mutated parental strains were crossed to generate the reciprocal hemizygote strains and diploid hybrids were selected in drug plates (300 ug/mL Hygromycin B, HphMx) and (100 ug/mL Nourseothricin). Finally, diploids were confirmed by *MAT* locus PCR^59^.

The different combinations of promoters and alleles were generated using *in vivo* assembly yeast recombinational cloning^60^,^61^. Briefly, the promoter and the allele selected for validation were amplified by PCR using a Phusion Flash High-Fidelity PCR Master Mix (Thermo scientific, USA). Additionally, the HphMx antibiotic resistance was also amplified by PCR and included in the genetics constructions; the overlap between PCR products was 50 bp. The PCR products were co-transformed with the linear plasmid pRS426 in the yeast strain BY4741 (*MATa*, *his3Δ1*, *leu2Δ0*, *LYS2*, *met15Δ0*, *ura3Δ0*). The circular plasmids generated in yeast were transferred to an *E. coli* DH5α strain and analysed by colony PCR under standard conditions. Three positives colonies containing the promoter, the allele and the HphMx cassette were selected for plasmid isolation and sequencing. The sequence identity of the promoters and alleles was analysed using the SGRP database BLAST service^25^,^26^. Finally, the parental strains were transformed with the complete genetics constructions, which were amplified by PCR using a Phusion Flash High-Fidelity PCR Master Mix (Thermo scientific, USA) and 70 bp primers for direct homologous recombination on the target locus, allowing the integration of the genetics constructions in the genome. The positives yeast colonies were analysed by colony PCR under standard conditions.

Oenological phenotypes were evaluated in sextuplicate in Synthetic Wine Must (MS300), prepared according to Rossignol *et al.*^62^. Briefly, MS300 was supplemented with a final concentration of 300 mgN/L of assimilable nitrogen (YAN) corresponding to 120 mgN/L of ammonium and 180 mgN/L of a mixture of 19 amino acids^56^. At the end of the fermentation (approximately at day 21, equivalent to a daily CO_2_ lost of less than 10% of the total CO_2_ lost across all 21 days), 12 mL of synthetic grape must (MS300) were centrifuged at 9000xg for 10 min and the supernatant was collected. 20 *u*L of MS300 were injected in a Shimadzu Prominence HPLC equipment and the concentration of glucose, fructose, glycerol, malic acid, acetic acid, succinic acid, ethanol, ammonium and each amino acid sources were measured using the HPLC analysis as previously described^63^.

### Luciferase expression

The genetic constructs carrying the luciferase reporter gene under the control of WE or NA *ASN1* promoters were assembled using yeast recombinational cloning, similar as it was described above. We used a previously described destabilized version of firefly luciferase allowing real-time quantification of gene expression in yeast^64^. With the aim to avoid effects of copy number and genetic context in gene expression, the genetic constructs were integrated in the *ASN1* locus. Additionally, the *UGA3* gene was deleted in the WE and NA strains carrying the luciferase constructs using *URA3* as selectable marker, similar as we previously described. The parental strains (WE and NA) carrying the luciferase constructs were analysed for luciferase expression using a Cytation3 microplate reader (Biotek, USA). Briefly, the strains were pre-grown in YPD, synthetic complete media (SC) and MS300 over-night, the cultures were diluted 1/100 to inoculate a 96 well plate with 200 uL of fresh culture media containing 0.1 mM of luciferin. The OD600nm and the luminescence of the cell cultures were monitored every 1-hour, all the experiments were performed in three biological replicas.

## Additional Information

**Accession Numbers:** The RNA-seq reads from this study are available from the NCBI’s Gene Expression Omnibus (GEO) under accession number GSE69115.

**How to cite this article**: Salinas, F. *et al.* Natural variation in non-coding regions underlying phenotypic diversity in budding yeast. *Sci. Rep.* **6**, 21849; doi: 10.1038/srep21849 (2016).

## Supplementary Material

Supplementary Information

Supplementary Dataset 1

Supplementary Dataset 2

Supplementary Dataset 3

Supplementary Dataset 4

Supplementary Dataset 5

## Figures and Tables

**Table 1:**
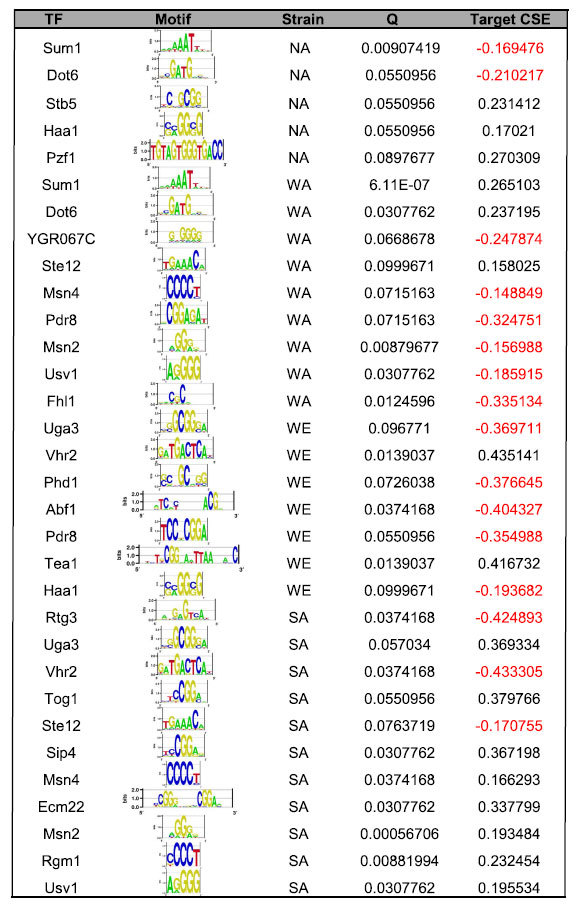
TFs whose motif instances significantly predict CSE.

The TF, its recognition motif, and the strain showing the difference in *trans*-regulation are as indicated. Q and Target CSE represent, respectively, the FDR-corrected p-values and differences in means (CSETarget - CSENon-target) in the comparison of CSE values between TF targets and non-targets.
