# Supplementary material for: Natural variation in non-coding regions underlying phenotypic diversity in budding yeast
Source: Sci Rep. 2016 Feb 22;6:21849. doi: 10.1038/srep21849 (PMC4761897; doi:10.1038/srep21849)

**Supplementary Information**

# Natural variation in non-coding regions underlying phenotypic diversity in budding yeast

Francisco Salinas[1§], Carl G. de Boer[2§], Valentina Abarca[3], Verónica García[3,4], Mara Cuevas[3], Sebastian Araos[3], Luis F. Larrondo[1], Claudio Martínez[3,4] and Francisco A. Cubillos[3]*

[1]Millennium Nucleus for Fungal Integrative and Synthetic Biology (MN-FISB), Departamento de Genética Molecular y Microbiología, Facultad de Ciencias Biológicas, Pontificia Universidad Católica de Chile, Casilla 114-D, Santiago, Chile

[2]Broad Institute of MIT and Harvard, Cambridge, MA, United States

[3]Centro de Estudios en Ciencia y Tecnología de Alimentos (CECTA), Universidad de Santiago de Chile (USACH), Santiago, Chile

[4]Departamento de Ciencia y Tecnología de los Alimentos, Universidad de Santiago de Chile (USACH), Santiago, Chile

* Corresponding author: francisco.cubillos.r@usach.cl

[§]These authors contributed equally

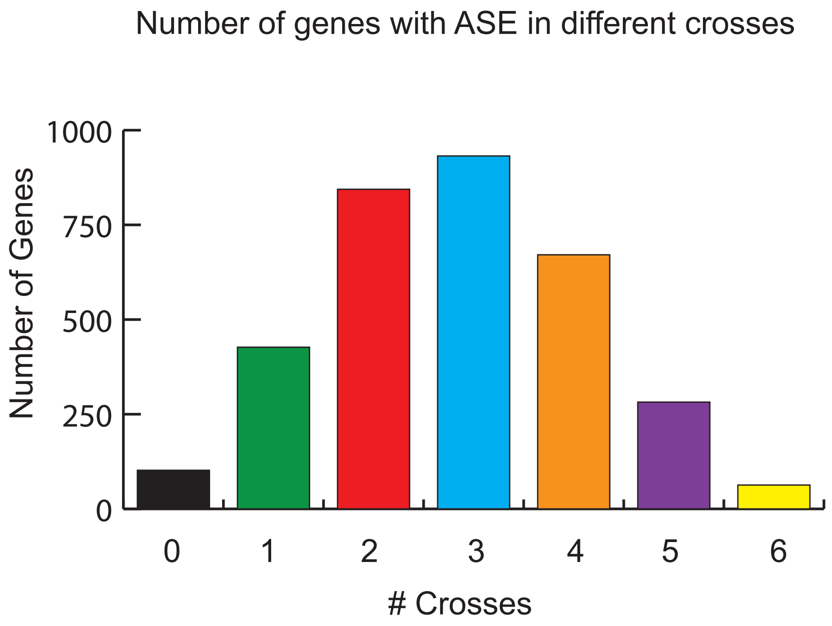
Number of genes with ASE in different crosses
1000
750
500
250
0
Number of Genes
0
1
2
3
4
5
6
# Crosses

Figure S1

SWM (MS300)

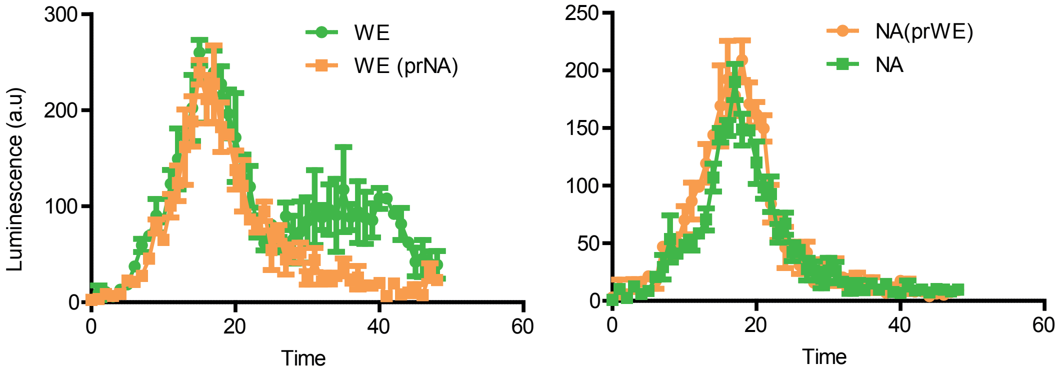


YPD

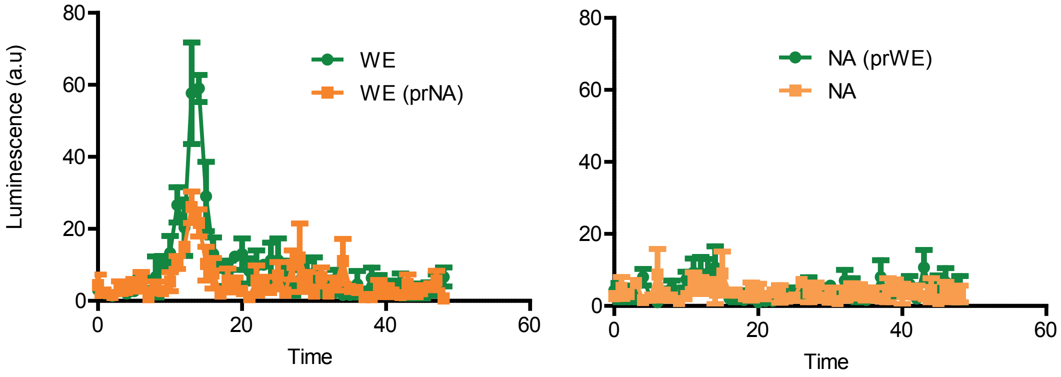

Supplement: Supplementary Information [file srep21849-s1.pdf]
